# Hospital Meetings

**Published:** 1895-11-23

**Authors:** 


### HOSPITAL MEETINGS.

### EAST LONDON HOSPITAL FOR CHILDREN.

At the half-yearly court of the governors of this hospital, which was held on Thursday, November 14th, under the chairmanship of Mr. Trinder, the Board of Management, in their report for the six months ending June 30th last, drew special attention to the important improvements in the hygienic conditions of the hospital which are now proceeding. The wards have been closed since October for this purpose, and will be re-opened, it is hoped, in January next, the conclusion of the work being contracted for by the fifth of that month.

The demand upon ward space has increased so largely since the present wards were opened in 1877, that the board recently wisely determined, having regard to the fact that the existing lavatories and bath-rooms had "become out of date and required renewal," to throw the space occupied by these into the wards and build out new offices on the most approved modern lines. The new bath-rooms, as explained by Mr. Trinder in presenting the report, will be at the entrance to the wards, so as to allow the little patients to be bathed before entering the ward at all, a particularly needful sanitary precaution in a hospital situated in such a district as Shadwell. The lavatories will be at the further end of the wards, cut off from them by thorough ventilation. The ventilation, too, of the wards generally, and the heating arrangements will undergo alteration and improvement; and the drainage, into the condition of which careful inquiry has been made by Dr. Dawson Williams and Mr. Horace Cheston, consulting architect to the hospital, will be put into a state of perfect soundness.

The nurses' quarters are also to be brought into line with the rest. The divisions between the present cubicles (which are of a very good size) will be carried up to the ceiling, and other improvements introduced. During the alterations the nursing staff are being housed outside the hospital.

Amongst other arrangements made since the issue of the last report, Miss Adelaide Row (formerly one of the Sisters of the hospital, and since lady superintendent of the Jenny Lind Infirmary, Norwich) has been appointed lady superintendent in succession to Miss F. A. Davies, resigned. Mrs. Fisher, who acted as matron and home sister for many years, has, with the concurrence of the governors, retired upon a pension, and the board of management was empowered at this last court to enter into similar arrangements in future in cases of long service, &c. The board would be well advised to follow the example of many other hospitals in this respect, and help their nurses in the most practical manner possible by assisting them to join the Royal National Pension Fund for Nurses.

With regard to funds, the contract price of the new works is £2,550, and of this the greater part is still to seek. The board are earnestly asking for even the smallest donations towards this end. The total receipts for the last half-year are considerably less than those for the corresponding period in 1894, which is accounted for by a decrease in legacies; but it is very satisfactory to note that there is an increase in both subscriptions and donations, a fact from which the board may well take comfort.

The report having been duly accepted, a vacancy among the trustees, caused by the death of Mr. Clifford Wigram, was filled by the unanimous election of Dr. Eustace Smith, and Mr. Charles Cheston was appointed to the post of treasurer, vacant by the retirement of Mr. Spence.

Very few governors were present at the court, these including Major R. Carr, Mr. Edmund Sharp, Rev. Father Gorman, Mr. Louis A. Dunn, Mr. J. Schwartz, Dr. J. Hayward, and the Rev. E. Bray.

### BRITISH HOME FOR INCURABLES, STREATHAM.

The half-yearly meeting of the governors of the British Home for Incurables took place at the Cannon Street Hotel on Thursday in last week. Mr. Bevan, who presided, alluded to the fact that at the last election pensioners only had been successful; now, as there had been one or two deaths in the home, and they were anxious to see it filled, they proposed to increase the number to be elected as in-patients that day to fifteen. This step would, of course, entail additional expense, but he trusted the public would come forward more liberally in the future than in the past. Large sums of money had recently been contributed to the Hospital Sunday Fund in the City, but in its benefits this charity did not participate. Another point to which he wished to draw attention was to the recent temporary establishment of a small seaside home at Margate. The cripples' nursery there having been given up, the place had been offered to them on very favourable terms for a year, and the experiment had succeeded admirably, many applications for admission having been received, and five annuitants were enjoying its advantages at the present time. Mr. Bevan held in his hand a letter signed by these annuitants testifying most gratefully to the benefit they were deriving from their stay. His hearers would be glad to learn that the Princess of Wales, the patron of the institution, had cordially approved of the plan. They must hope to be able in the future to build a proper home. As the ultimate result of the poll which followed, 15 candidates were elected as in-patients and seven to pensions. Spectators of the unseemly trafficking in votes which takes place on these occasions, and which must be inseparable from such a system, may well wonder how much longer it will be tolerated in these days of greater enlightenment in the management of charities. Like subscribers' letters, another example of the pernicious principle of giving in order to get rather than from the pure pleasure of being helpful, it must be abolished when people learn, as they surely will, to understand the real privilege of giving; but the time seems slow in coming. One may, however, urge the friends of those institutions where the voting system still obtains to sit down and consider the matter for themselves—the unjustifiability of allowing interest to overpower justice—and to set about instituting this crying reform with all the promptitude possible.

